# Half of Prescribed Antibiotics Are Not Needed: A Pharmacist-Led Antimicrobial Stewardship Intervention and Clinical Outcomes in a Referral Hospital in Ethiopia

**DOI:** 10.3389/fpubh.2020.00109

**Published:** 2020-04-09

**Authors:** Gebremedhin Beedemariam Gebretekle, Damen Haile Mariam, Workeabeba Abebe Taye, Atalay Mulu Fentie, Wondwossen Amogne Degu, Tinsae Alemayehu, Temesgen Beyene, Michael Libman, Teferi Gedif Fenta, Cedric P. Yansouni, Makeda Semret

**Affiliations:** ^1^School of Pharmacy, Addis Ababa University, Addis Ababa, Ethiopia; ^2^School of Public Health, Addis Ababa University, Addis Ababa, Ethiopia; ^3^School of Medicine, Addis Ababa University, Addis Ababa, Ethiopia; ^4^J. D. MacLean Centre for Tropical Diseases and McGill University, Montreal, QC, Canada

**Keywords:** AMR, antimicrobial stewardship, antimicrobial prescribing, audit-feedback, pharmacist, Ethiopia, LMIC, LRS

## Abstract

Intense antibiotic consumption in Low- and Middle-Income Countries (LMICs) is fueled by critical gaps in laboratory infrastructure and entrenched syndromic management of infectious syndromes. Few data inform the achievability and impact of antimicrobial stewardship interventions, particularly in Sub-Saharan Africa. Our goal was to demonstrate the feasibility of a pharmacist-led laboratory-supported intervention at Tikur Anbessa Specialized Hospital in Addis Ababa, Ethiopia, and report on antimicrobial use and clinical outcomes associated with the intervention.

**Methods:** This was a single-center prospective quasi-experimental study conducted in two phases: (i) an intervention phase (November 2017 to August 2018), during which we implemented weekly audit and immediate (verbal and written) feedback sessions on antibiotic prescriptions of patients admitted in 2 pediatric and 2 adult medicine wards, and (ii) a post-intervention phase (September 2018 to January 2019) during which we audited antibiotic prescriptions but provided no feedback to the treating teams. The intervention was conducted by an AMS team consisting of 4 clinical pharmacists (one trained in AMS) and one ID specialist. Our primary outcome was antimicrobial utilization (measured as days of therapy (DOT) per 1,000 patient-days and duration of antibiotic treatment courses); secondary outcomes were length of hospital stay and in-hospital all-cause mortality. A multivariable logistic regression model was used to explore factors associated with all-cause in-hospital mortality.

**Results:** We collected data on 1,109 individual patients (707 during the intervention and 402 in the post-intervention periods). Ceftriaxone, vancomycin, cefepime, meropenem, and metronidazole were the most commonly prescribed antibiotics; 96% of the recommendations made by the AMS team were accepted. The AMS team recommended to discontinue antibiotic therapy in 54% of cases during the intervention period. Once the intervention ceased, total antimicrobial use increased by 51.6% and mean duration of treatment by 4.1 days/patient. Mean LOS stay as well as crude mortality also increased significantly in the post-intervention phase (LOS: 24.1 days vs. 19.8 days; in hospital death 14.7 vs. 6.9%). The difference in mortality remained significant after adjusting for potential confounders.

**Conclusions:** A pharmacist-led AMS intervention focused on duration of antibiotic treatment was feasible and had good acceptability in our setting. Cessation of audit-feedback activities was associated with immediate and sustained increases in antibiotic consumption reflecting a rapid return to baseline (pre-intervention) prescribing practices, and worse clinical outcomes (increased length of stay and in-hospital mortality). Pharmacist-led audit-feedback activities can effectively reduce antimicrobial consumption and result in better-quality care, but require organizational leadership's commitment for sustainable benefits.

## Introduction

The association between Antimicrobial Resistance (AMR) and antibiotic consumption has been well documented (1–3). The global increase in antibiotic consumption noted in the past 15 years has predominantly been driven by Low- and Middle-income countries (LMICs) (4), which can least afford the tremendous human and economic costs of AMR (5, 6). While large segments of populations still lack access to essential antimicrobials, patients hospitalized in low-resource settings (LRS) tend to be on multiple, broad-spectrum antibiotics continuously throughout their healthcare journey. Central to the issue of antibiotic overconsumption in LRS hospitals is the lack of diagnostic infrastructure and severe gaps in infection prevention and control—leading to a widespread culture of prescribing antibiotics both empirically and prophylactically.

We recently implemented a laboratory bundle intervention aimed at improving bacteriology services in Ethiopia's largest teaching and referral hospital, which currently contributes a significant proportion of the national AMR surveillance data (7). In the first year after implementation, we reported widespread resistance of gram-negative bacteria to locally available antibiotics including carbapenems, a class of antibiotics which had been introduced in Ethiopia only 3 years prior (8). In parallel, antibiotic expenditures in the institution escalated (20% increase compared to the year prior) with meropenem and vancomycin accounting for over 40% of the total institutional antibiotic budget. Although a national AMR action plan and an antimicrobial stewardship implementation guide were developed for Ethiopia (9, 10), “implementation readiness” in individual hospitals, including in tertiary care urban referral centers, remains extremely low. Gaps in health system processes, lack of sufficiently trained personnel and competing priority initiatives for the institutions' leadership, present formidable barriers to delivering functional stewardship programs in Ethiopia.

Studies conducted in LMICs have shown that models of stewardship using non-specialized health care providers such as clinical pharmacists can lead to reductions in antimicrobial consumption (11–13). However, clinical pharmacists play a predominantly dispensing role in most LRS hospitals and hardly ever interact with physician members of the treating teams—remaining unknown entities in terms of potential contribution to clinical decision-making (14). Physicians and other professionals frequently raise concerns regarding the applicability of antimicrobial stewardship particularly for tertiary care hospitals where patients are at significant risk of hospital-associated multi-drug resistant infections.

We assessed the feasibility and impact of a pharmacist-led intervention on antibiotic consumption and on clinical outcomes, in a tertiary care referral hospital in Ethiopia with a significant burden of Hospital-associated infections (HAI) and AMR. We used a quasi-experimental study design comparing outcomes during and after the intervention, to determine whether cessation of audit-feedback activities would lead to changes (immediate or gradual) in outcomes.

## Methods

### Study Setting

The study was conducted between November 2017 and January 2019 in Tikur Anbessa Specialized Hospital (TASH), the largest referral and teaching hospital in Ethiopia and the site of an ongoing study on HAI funded by and in collaboration with the Research Institute of the McGill University Health Centre. The hospital has 800 operational beds, of which 191 are dedicated to pediatric services, and provides tertiary medical, surgical and obstetrical care to 20,000 inpatients and 330,000 outpatients per year. Average length of stay is 9.3 days, and in-patient mortality for the year preceding our study was reported as 5.8%. The hospital is staffed with 1059 physicians (residents-in-training and senior faculty) of which 4 had expertise in Infectious Diseases at the time of the study; 80 pharmacists and 7 microbiology laboratory technologists.

The study was conducted on 2 general medicine wards (total capacity of 61 beds) and 2 pediatric wards (66 beds).

### Study Design and Ethics

This was a prospective, quasi-experimental study assessing the impact of a weekly audit-feedback intervention on antibiotic consumption and clinical outcomes. The study population was patients hospitalized in the selected wards and receiving any systemic antibiotics on audit days. The study was approved by the Institutional Review Board (IRB) of the College of Health Sciences, Addis Ababa University (Protocol Number: 045/15/IM), and the Research Institute of the McGill University Health Centre.

### Pre-intervention Structural Activities

#### Guidelines and Cumulative Antibiogram

We developed institutional guidelines for the empiric management of the 4 most common indications for antibiotic therapy in the institution: Sepsis, Febrile Neutropenia, Community-Acquired and Hospital-Associated Pneumonia. Aggregate data collected from the microbiology laboratory over the preceding year were used to develop an institutional cumulative antibiogram. Treatment guidelines and cumulative antibiogram were uploaded into a commercially available customizable stewardship app (the Sanford guide with Stewardship Assist, Antimicrobial Therapy, Inc, USA). Prescribers and pharmacists were given free access to this tool on their mobile devices for a period of 3 years (longer than the duration of the study).

#### Training of Pharmacists, Information Sessions for Physicians

We provided 4 half-day case-based interactive training sessions (over a period of 2 weeks) to the clinical pharmacists on basic concepts of antibiotic therapy, with a focus on acceptable *duration of treatment* for common syndromes. They were further introduced to the microbiology laboratory and given basic training on laboratory report interpretation. We also conducted 30-minute information sessions for clinicians to explain the rationale, benefits, and limitations of stewardship interventions, emphasizing that antimicrobial stewardship (AMS) recommendations were not medical orders and had to be reviewed and approved by treating teams. We further encouraged treating team members to participate in the weekly AMS sessions.

### Audit-Feedback Intervention

The intervention was conducted between November 2017 and August 2018, for a period of 10 months (40 weeks). AMS team rounds were carried out weekly (every Tuesday 2–4 p.m. for pediatric wards, and every Thursday 2–4 p.m. for adult medicine wards) during the 40 weeks of the intervention period.

#### Team Composition and Roles

The AMS team was composed of 4 pharmacists (led by GBG), 3 Ethiopian physicians certified in Infectious Diseases alternating weekly, and one ID physician-in-training. Three Canadian physicians specialized in ID and clinical microbiology provided training of pharmacists, information sessions for physicians, and oversaw the development of the institutional cumulative antibiogram and treatment guidelines. They additionally supervised 4 audit-feedback sessions every 3 months, for a total of 12 sessions.

#### Antibiotic Targets

All systemic antibiotics (oral and parenteral) prescribed and dispensed in hospital on the selected wards were included; antimicrobials used to treat parasitic, viral, or mycobacterial infections were excluded. We aimed to audit between 8 and 10 charts in medicine and in pediatrics each, for a total of 16–20 charts per week. Given previous point-prevalence estimates that 80% of patients hospitalized in medical and pediatric wards (100 patients in total) are on antibiotics on any given day (*unpublished data)*, we estimated that 20 audits/week would represent 25% of patients receiving antibiotics on these wards.

#### Audit and Feedback

Pharmacist members of the AMS team approached treating teams on the selected wards to identify patients receiving any of the targeted antibiotics on audit days, and whose discharge from hospital was not anticipated for at least 3 days. Through chart reviews supplemented with information from treating teams, they prepared a narrative summary of the case for discussion with the AMS team, focusing on the start date of therapy, indication for therapy, whether the patients had had microbiological workup, and results of testing.

The hospital has currently no automatic reporting or delivery mechanism for microbiology results. Generally, clinicians (junior house-staff) collect results directly from the laboratory. During the intervention, if results of testing were not yet known to the treating team, pharmacists would go to the laboratory on audit days to check for results.

Each case presentation was followed by a short team discussion, centered on findings of the diagnostic workup and on acceptable duration of therapy. Members of the treating team were invited to contribute to the discussion. After reaching a consensus, the AMS team issued a recommendation which was transmitted verbally to the treating team and also documented on a structured form attached to the patient chart (Supplementary Data Sheet 1). When consensus could not be reached, the recommendation of the ID physician prevailed.

Recommendations were broadly categorized into four groups: (1) discontinue antibiotic, (2) change (dose, duration, route and/or antibiotic type), (3) continue with specified duration, and (4) consult ID service. Pharmacists followed-up within 24 to 48 h to assess acceptance/non-acceptance of recommendations. Reasons for non-acceptance were documented.

### Post-intervention Phase

The post intervention phase was from September 2018 to January 2019. During this period, 2 pharmacists reviewed medical charts of patients receiving any of the targeted antibiotics, at a similar frequency as during the intervention phase, and using similar criteria as during the intervention (patients whose discharge was not anticipated for the next 3 days). They collected audit data but did not provide structured feedback to prescribers and AMS rounds were not held. Only data available on the medical chart was collected by the auditors; if microbiology laboratory results were not known, the auditors did not make specific efforts to collect results from the laboratory. Prescribers continued to have access to treatment guidelines and the cumulative antibiogram on their mobile devices.

### Data Collection

Data elements for intervention and post-intervention phases included demographic and clinical characteristics (comorbidities and main diagnosis at admission), antibiotic dosage and start dates, and documented indications for antibiotics. When these could not be ascertained from chart review or from discussion with treating teams, they were considered to be “empiric/source of infection undocumented.” During the intervention phase, AMS team recommendations and acceptance or non-acceptance of the recommendations were collected. For both intervention and post-intervention phases, antibiotic end dates, date of discharge from hospital or date of death if occurred in hospital were also collected. Data was entered into an electronic data capture instrument (Supplementary Data Sheet 2).

### Outcome Measures

The primary outcome of our study was antimicrobial utilization, expressed in treatment duration and days of therapy (DOT) per 1,000 patient-days. We defined duration of treatment as the number of consecutive days during which a patient received a specific antibiotic, and DOT as the aggregated sum of all the days during which a patient received any antibiotic. For patients on multiple antibiotics, we calculated the sum of DOTs for each antibiotic received (regardless of dose or route). We compared antibiotic treatment duration and DOT during and post-intervention for our study population.

Our secondary outcomes were length of hospital stay (LOS) and all-cause in-hospital mortality during the study periods. These were also compared during and post-intervention.

### Statistical Analysis

Patients baseline and hospital stay characteristics during and post-intervention periods were compared using Fisher's exact or χ2 tests for categorical variables, and *t*-test for continuous variables. Data on antibiotic utilization were aggregated into months (10 months intervention and 5 months post-intervention); differences in mean duration of treatment and DOT per 1,000 patient-days during the 2 periods were compared using *t*-test.

We performed univariate and multivariable logistic regressions to assess the association of certain parameters on the pre-specified secondary outcome, in-hospital mortality. Adjustments were made for age, known comorbidities, febrile neutropenia, and suspected HAI. Further subgroup mortality analyses were performed for those with specific comorbidities (malignancy and liver disease).

All tests were conducted at 2-sided 0.05 level of significance. All statistical analyses were performed on SPSS (IBM SPSS Statistics for Windows, version 25.0; IBM Corp., Armonk, N.Y., USA).

## Results

### Study Population

We audited a total of 1264 prescriptions from 707 individual patients during the intervention phase, and 1,138 prescriptions from 402 patients in the post-intervention phase, for an average of 18 chart reviews per week over the duration of the study. The most commonly reported indications for antibiotics during both periods were infections of respiratory source (Table 1). A greater proportion of patients during the intervention phase were prescribed antibiotics without a documented source compared to the post-intervention phase (25 vs. 16%) and were also categorized as having a HAI (78 vs. 66%). A greater proportion of patients in the post-intervention phase received antibiotics for “febrile neutropenia” and had prior comorbidities including malignancy.

**Table 1:** Demographic and clinical characteristics of patients during intervention and post-intervention periods.

**Variable**	**Intervention period *n* (%) *N* = 707**	**Post-intervention period *n* (%) *N* = 402**	***p***
Gender male	381 (53.9)	233 (58.0)	0.19
**Age category**			
Age <2 years	266 (37.6)	127 (31.6)	0.13
Age 2–16 years	143 (20.2)	87 (21.6)	
Adult (>16 years)	298 (42.1)	188 (46.8)	
**Admission ward**			
Pediatrics ward	400 (56.6)	204 (50.7)	0.07
Medicine ward	307 (43.4)	198 (49.3)	
Presumed infection at admission	425 (60.1)	270 (67.2)	0.26
Hospital-associated infection suspected	554 (78.4)	267 (66.4)	<0.001*
**Indication for antibiotic**			
Respiratory infection	233 (32.9)	158 (39.3)	0.12
Neurologic infection	111 (15.7)	75 (18.7)	0.21
Genitourinary infection	41 (5.8)	25 (6.2)	0.78
Gastrointestinal infection	39 (5.5)	29 (7.2)	0.26
Late onset neonatal sepsis	36 (5.1)	24 (6.0)	0.53
Febrile neutropenia	29 (4.1)	30 (7.5)	0.02*
Skin/soft tissue infection	19 (2.7)	15 (3.7)	0.33
Cardiovascular infection	15 (2.1)	10 (2.5)	0.69
Musculoskeletal infection	12 (1.7)	5 (1.2)	0.56
Surgical site infection	4 (0.6)	8 (2.0)	0.06
Others	18 (2.5)	16 (3.9)	0.87
Empiric/source undocumented	178 (25.2)	66 (16.4)	0.001*
Known prior comorbidities (any)	364 (51.5)	251 (62.4)	0.001*
Liver disease	39 (5.5)	11 (2.7)	0.001*
Renal disease	106 (15.0)	53 (13.2)	0.26
Malignancy	134 (19.0)	96 (23.9)	0.03*
HIV/AIDS	28 (4.0)	19 (4.7)	0.06
Diabetes mellitus	47 (6.7)	42 (10.4)	0.047*
Cardiovascular diseases	75 (10.6)	73 (18.2)	0.13
Number of antibiotics/patient (Mean ± SD)	1.78 ± 0.7	2.83 ± 1.2	0.001*
Hospital stay (days) (mean ± SD)	19.8 ± 12	24.1 ± 13.9	<0.001*
Death in hospital *n*(%)	49 (6.9%)	59 (14.7)	<0.01*

* *Statistically significant*.

### Audit-Feedback Results

Of the 1,264 prescriptions audited during the intervention phase, the AMS team recommended to discontinue antibiotic in 685 (54.2%). The most common justification (52% of cases) for this recommendation was sufficient treatment duration. The majority (96.4%) of recommendations were accepted by the treating teams (Table 2).

**Table 2:** AMS team recommendations and prescribers' acceptance of recommendation.

**Variables**	***n* (%)**
**AMS recommendations (*****n*** **= 1264 prescriptions, 707 patients)**	
Discontinue current antibiotic	685 (54.2)
Continue current antibiotic	477 (37.7)
Change current antibiotic (dose/frequency/route)	102 (8.1)
Start an alternate antibiotic(s)^a^	59 (8.3)
Consult ID^a^	32 (4.5)
**Reasons for discontinuing current antibiotic**^**b**^ **(*****n*** **= 685)**	
Treatment duration sufficient	359 (52.4)
Indication/source not evident by history	148 (21.6)
Investigations do not support diagnosis/source of infection	117 (17.1)
Spectrum inappropriate	65 (9.5)
Septic workup insufficient for suspected focus	59 (8.6)
**Reasons for changing current antibiotic (*****n*** **= 102)**	
Dosing inappropriate	80 (78.4)
Route inappropriate	11 (10.8)
Safety consideration	4 (3.9)
Cost/availability	7 (6.9)
Overall acceptance of recommendations (*n* = 1264)	1219 (96.4)
**Reasons for non-acceptance of recommendations (*****n*** **= 45)**	
Patient nearing the end of planned therapy	26 (2.1)
Suspicion of an additional focus or pathogen	11 (0.9)
Suspicion of antibiotic resistance	8 (0.6)

a *Percentage is calculated using number of prescriptions as denominator*.

b *More than one reason to discontinue antibiotics was given in 63 cases, therefore the % don't add up to 100%*.

The most commonly prescribed antibiotics were third-generation cephalosporins (3GC) followed by vancomycin and cefepime during the intervention phase. Once the intervention ceased, meropenem became the third most commonly prescribed antibiotics after 3GC and vancomycin. Narrow-spectrum antibiotics particularly those targeting gram-positive organisms (cloxacillin, cefazolin) were rarely prescribed (Table 3). The AMS team recommendation to discontinue antibiotics applied across all antibiotics, with over 50% of almost every type of antibiotic being deemed unnecessary at the time of audit (Table 3).

**Table 3:** Patterns of prescriptions and recommendations to discontinue specific antibiotics during the intervention, and patterns of prescriptions during the post-intervention phases.

	**Intervention**		**Post-intervention**
**Name of Antibiotic**	**Total number of prescriptions (*n* = 1264); (%)**	*Recommendation to discontinue (*n* = 685); (%)	**Total number of prescriptions (*n* = 1141); (%)**
Ceftriaxone/Cefotaxime	376 (29.7)	190 (50.5)	284 (24.9)
Vancomycin	235 (18.6)	141 (60)	191 (16.7)
Cefepime	120 (9.5)	75 (62.5)	111 (9.7)
Metronidazole	110 (8.7)	65 (59.1)	113 (9.9)
Ampicillin	86 (6.8)	44 (51.2)	59 (5.2)
Meropenem	83 (6.6)	32 (38.6)	122 (10.7)
Gentamycin	67 (5.3)	40 (59.7)	67 (5.9)
Ceftazidime	40 (3.2)	27 (67.5)	43 (3.8)
Piperacillin-tazobactam	29 (2.3)	9 (31)	9 (0.8)
Azithromycin	28 (2.2)	17 (60.7)	50 (4.4)
Ciprofloxacin	26 (2.1)	17 (65.4)	36 (3.2)
Ampicillin-Sulbactam	24 (1.9)	10 (41.7)	14 (1.2)
Cloxacillin	15 (1.2)	10 (66.7)	12 (1.1)
Clindamycin	14 (1.1)	5 (35.7)	9 (0.8)
Cefazolin	4 (0.3)	0	3 (0.3)
Others	7 (0.6)	3 (42.9)	18 (1.6)

* *Proportion calculated as the % discontinued for each antibiotic*.

### Primary Outcomes

#### Duration of Treatment

**Figure 1:**
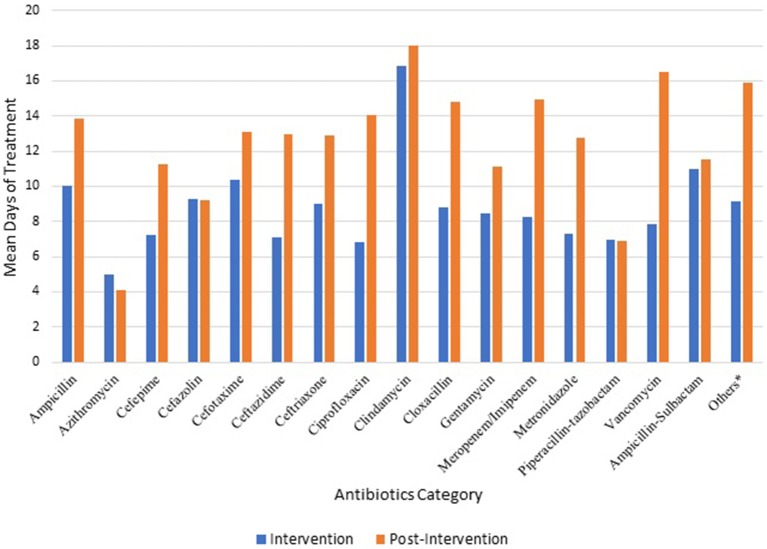
Average treatment duration (per patient) during- and post-intervention phases.

The average duration of antibiotic treatment increased significantly for all antibiotics, from 8.7 ± 6.9 days during the intervention, to 12.8 ± 11.7 days in the post-intervention phase (*p* = 0.002). The increase in duration was noted for all antibiotics with the exception of azithromycin and combination beta-lactamase inhibitor antibiotics, both of which were prescribed only in a small number of cases (Figure 1).

**Figure 2:**
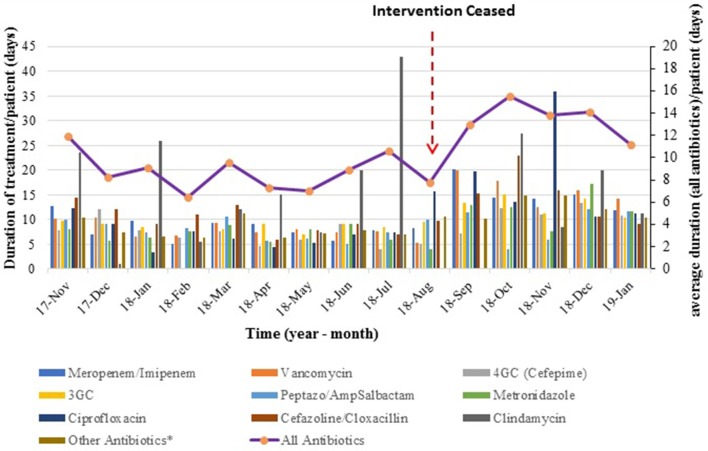
Average duration of antibiotic therapy over time. The bar graph represents monthly average duration (per patient) for each antibiotic type, while the line graph shows the mean duration of treatment for all antibiotics combined.

Trends for average duration of treatment over time show that duration of treatment was stable (flat line) during the 10 months of the intervention period, rose very sharply once the intervention ceased and reached a plateau (Figure 2).

#### DOT

**Figure 3:**
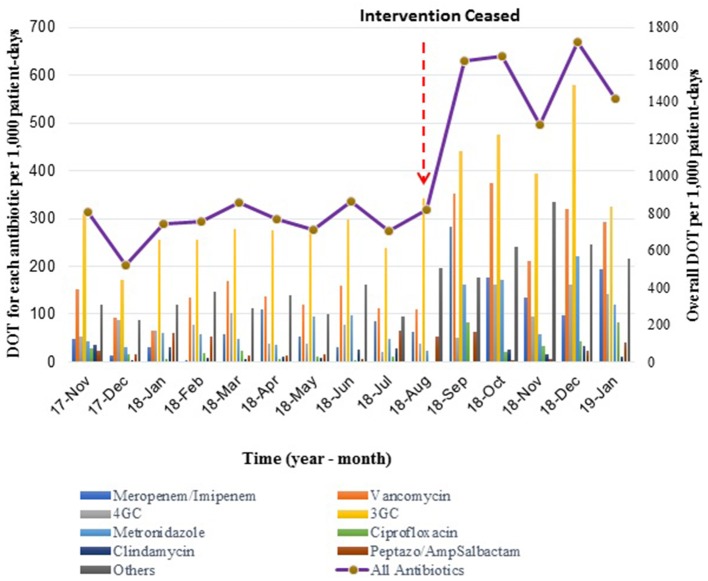
Mean Days of Therapy (DOT) per 1,000 patient-days during- and post-intervention phases. The bar graph shows monthly DOT for each type of antibiotic, while the line graph represents the overall monthly DOT per 1,000 patient-days.

There was a 2-fold increase in DOT/1000 patient days once the intervention ceased, from a mean DOT of 754 ± 99.8/1000 patient-days in the intervention phase to 1549 ± 175.2/1000 patient-days during the following 5 months. Trends of DOT/1000 patient-days over time showed a similar pattern to durations of treatment: stable rates during the intervention, followed by a sharp increase and a new plateau (Figure 3).

### Secondary Outcomes

Length of stay in hospital increased from 19.8 ± 12.0 days during the intervention period, to 24.1 ± 13.9 days once the intervention ceased (20% increase in duration, *p* < 0.001). Additionally, we note that all-cause in-hospital mortality increased from 6.9% during the intervention to 14.7% post-intervention (*p* < 0.01). Variables associated with in-hospital death for our study population are shown in Table 4. Age older than 16 years, febrile neutropenia as the main indication for antibiotic therapy, and pre-existing comorbidities were significantly correlated with death in the univariate analysis. In the multivariate analysis, underlying malignancy and liver disease (but not HIV or renal disease) significantly increased the risk of death (OR 2.1, 95% CI: 1; 5.3 and OR 2.1; 95% CI 1; 3.2, respectively). Febrile neutropenia and age older than 16 also remained significant risk factors for mortality in-hospital. Even after adjusting for these confounding variables, the difference in mortality between intervention and post-intervention periods remained significant with an adjusted odds ratio of 2.3 (95% CI 1.5, 3.5).

**Table 4:** All-cause in-hospital mortality during and after the intervention.

**Variables**	**Status at discharge** ***n*** **(%)**		**Odds Ratio (95%CI)**	
	**Alive *N* = 1001**	**Dead *N* = 108**	**COR (95%CI)**	**AOR (95%CI)**
**Phases/time period**				
Intervention Phase	658 (65.7)	49 (45.4)	1.00	1.00
Post-intervention	343 (34.3)	59 (54.6)	2.31 (1.55, 3.45)*	2.32 (1.53, 3.53)*
Age (in years), ≥16 years old	416 (41.6)	70 (64.8)	2.5 (1.71, 3.92)*	2.30 (1.42, 3.74)*
Gender, Male	556 (55.5)	58 (53.7)	0.93 (0.62, 1.38)	
Infection at admission	631 (63.0)	64 (59.3)	0.85 (0.57, 1.28)	
Infection suspected/confirmed	775 (77.4)	90 (83.3)	1.45 (0.86, 2.44)	
Indication Febrile Neutropenia	45 (4.5)	14 (13.0)	3.16 (1.68, 5.97)*	2.39 (1.12, 5.13)*
Suspected HAI	745 (74.4)	76 (70.4)	1.22 (0.79, 1.88)	
Prior comorbidities (Any)	538 (53.7)	77 (71.3)	2.14 (1.38, 3.30)*	1.12 (0.64, 1.98)
Liver disease	40 (4.0)	10 (9.3)	2.45 (1.19, 5.05)*	2.15 (1.13, 5.33)*
Renal disease	134 (13.4)	25 (23.1)	1.95 (1.20, 3.16)*	1.73 (0.99, 3.01)
Malignancy	199 (19.9)	31 (28.7)	1.62 (1.04, 2.53)*	1.34 (1.04, 3.18)*
HIV	39 (3.9)	8 (7.4)	1.97 (0.89, 4.34)	
Diabetes mellitus	77 (7.7)	12 (10.1)	1.26 (0.79, 1.99)	

*COR, crude Odds ratio; AOR, Adjusted Odds ratio; HAI, Hospital-Associated Infection*;

* *statistically significant*.

## Discussion

Prescribing broad-spectrum antibiotics empirically and for prolonged duration is a common and enduring practice in many LRS. In Ethiopia, up to 85% of hospitalized patients are on antibiotics on any given day (15). Further, prescriptions tend to follow patterns whereby the latest antibiotics introduced into the country gradually replace previous generations of broad-spectrum antibiotics, which themselves replaced first-line agents only a few years prior. In our institution, spiraling costs due to increasing consumption of 3 specific antibiotics (meropenem, vancomycin, and cefepime) prompted the institution's leadership to consider urgent implementation of an antimicrobial stewardship intervention. When faced with such pervasive use of antibiotics, adopting “restrictive” policies would be the most expedient particularly from a cost-reduction perspective—but could have dire consequences in a setting where bacterial sepsis is common, and expertise in infectious diseases is rare. On the other hand “enabling” interventions, while effective in settings with expertise and resources, are more challenging to implement and sustain in LRS—especially when bacterial culture and antimicrobial susceptibiltiy testing are unavailable or underutilized (16–18).

We offer in this study a proof-of-principle that a pharmacist-led intervention on wards where patients receive complex medical care (including management of malignancy) was well received and beneficial. We embedded audit-feedback activities within existing structures and involved members of the treating team (housestaff). We ensured treating teams maintained prescription autonomy. We provided modest structural and educational support through an academic collaboration with McGill University, leveraging external clinical microbiology expertise to build local capacity. The presence of one infectious diseases specialist per audit-feedback session to supervise and approve recommendations facilitated implementation and acceptability.

The majority of study patients were receiving antibiotics for a suspected HAI. By focusing most of our feedback on duration of treatment, we were able to recommend discontinuing antibiotics in over 50% of cases. Since the wards we targeted were the main consumers of costly antibiotics, an intervention limited to only 25% of patients on just 2 wards resulted in substantial cost savings for the entire institution: antibiotic costs decreased by an amount equivalent to 35,000 USD (19% reduction compared to the year preceding the intervention)—equivalent to the total cost of laboratory reagents invested in support of the bacteriology laboratory (7).

One of the strengths of our study is that we analyzed DOT rather than Daily Defined Dose (DDD); the latter would have been difficult to interpret in a population that includes children, and would have provided a biased view of antibiotic consumption in a setting where combination therapy is very popular (the average patient is prescribed 2 antibiotics simultaneously). Cessation of audit-feedback activities resulted in an immediate 2-fold increase in antibiotic utilization (duration of treatment and DOT/1000 patient days), and was most marked for meropenem (74% increase post intervention), vancomycin (61%), and cefepime (53%). This suggests that clinicians had acquired better knowledge of the burden of drug-resistance, through access to the cumulative institutional antibiogram which revealed widespread resistance of gram-negative organisms to 3rd generation cephalosporins. It is therefore not suprising they prescribed more “last resort” drugs. In the absence of immediate feedback, they would opt for prolonged treatment durations as the “safer” option.

Cessation of weekly feedback sessions was also associated with an increase in hospital length of stay compared to the intervention period (from 19 to 24 days, representing a 20% increase) – a meaningful difference for the targeted wards, which at baseline have substantially longer lengths of stay than the institutional average of 9 days. Audit-feedback interventions in high-resource settings have reported an average reduction in length of stay of 1.12 days (19). The more dramatic increase in length of stay noted post-intervention in our setting is likely explained by the fact that patients remain in hospital *to* receive intravenous antibiotics. It is therefore reasonable to suggest that cessation of audit-feedback activities had a *direct* impact on length of stay, highlighting the need for sustained efforts.

The tremendous variability inherent in many studies on antimicrobial stewardship limits interpretation of mortality benefits attributable to specific interventions (4, 20–22), but pharmacist-led interventions in China and in Brazil have been associated with a 10% reduction in mortality (23, 24). In our study, all cause in-hospital mortality was 2-fold higher post-intervention compared to the intervention phase. While a higher proportion of patients in the post-intervention phase had comorbidities (notably malignancy and liver disease) placing them at higher risk of death in-hospital, the difference in mortality between the 2 periods remained significant even after adjusting for these variables—suggesting that audit-feedback activities may indeed have had a beneficial impact on patient outcomes.

We know from a recent surveillance study on HAI conducted in the same institution that patients with infection tend to have poor outcomes, with an overall mortality rate from HAI of 20%. (8). Therefore, the 15% mortality noted in the post-intervention phase probably reflects a return to “baseline” mortality rates of patients with suspected infection in this institution, while the 7% mortality observed during the intervention was significantly lower than expected, in spite of reductions in antibiotic utilization. There are 2 plausible explanations for the beneficial effect of the intervention. First, the infectious diseases specialist's input was critical to clarify specific terms and indications for treatment. Studies conducted in high-income settings have convincingly demonstrated that infectious diseases intervention is associated with improved patient outcomes including reduced mortality and length of stay (25). In our setting, diagnoses such as “febrile neutropenia” were frequently invoqued by treating teams as an indication for antibiotics even if fever and neutropenia were not documented. Likewise, diagnoses of “pneumonia” were often presumptive and not confirmed by X-rays; diagnoses of “endocarditis” were made without positive blood cultures or other accepted criteria for endocarditis. The infectious diseases specialist played a key role in terms of clarifying diagnoses, which ultimately may have led to better management of infectious cases. Cessation of the audit-feedback sessions most certainly led to a drop in the accuracy of treatment indications, quality of documentation, and possibly frequency of microbiologic testing—leading in turn to more empiricism, longer treatment courses, longer lengths of stay and possibly increased mortality. Second, communication with the laboratory was noticeably better during the intervention, compared with pre-intervention or post-intervention periods. Failure to communicate critical bacteriology results is a problem that has been recognized in many LRS (26, 27). During the intervention, clinical pharmacists made some effort to seek microbiology results when these were not known to treating teams. Results were then discussed during audit-feedback sessions with the infectious disease specialist. The responsibility of collecting laboratory results (and acting upon them) would have reverted back to members of the treating teams post-intervention, and would have been entirely dependent on individual effort—possibly contributing to excess mortality. Our experience therefore suggests that infection experts and pharmacists in LRS can play a significant role in palliating some of the communication deficiencies between laboratory and clinician, and in optimizing management of patients with suspected infections.

Our study has several limitations. First, selection bias and lack of controls could have had a confounding effect on outcomes. We made efforts to limit our exclusion criteria only to patients whose discharge from hospital was imminent, and during both periods performed audits on cases identified by treating teams. It is possible that treating teams unknowingly directed the auditors toward the sickest patients in the post-intervention period, but more likely they would have done so *during* the intervention, knowing the infection specialist would be present to provide input. The higher proportion of patients with comorbidities post-intervention is therefore probably more reflective of secular trends in admission. In any case, whether or not the excess mortality post-intervention can be partially attributed to cessation of audit-feedback is less relevant than the fact mortality during the intervention was no higher than the historical institutional mortality rate. Indeed, antimicrobial stewardship is generally not expected to improve clinical outcomes but rather to reduce collateral damage without changing outcomes (19, 28). Our findings clearly show that cessation of over half the antibiotic prescriptions certainly had no detrimental effect on care, in fact might have been clearly beneficial.

Assessing outcomes during 3 time periods, namely pre- during and post-intervention, would have provided a more optimal assessment of the intervention's impact over time. This was not possible in the context we were operating under, notably the risk that overly restrictive policies would be implemented before we could demonstrate feasibility and effectiveness of “*enabling* interventions”. Also, the relatively small number of data points during and after the intervention made it difficult to perform a formal interrupted time-series (ITS) analysis, a stronger design for causal inference in quasi-experimental studies (19, 29). ITS analysis would have required significantly longer follow-up post intervention, which was neither practical nor entirely ethical considering the now well-recognized benefits of stewardship interventions.

Finally, this was a single center study conducted in a tertiary teaching hospital; hence our specific approach may not be applicable to smaller hospitals in LRS with even fewer infection specialists. In our experience the infection specialists played a key role in terms of providing credibility to the AMS team, interpreting available clinical data and assessing the safety of discontinuing antibiotics based on patients' clinical course. Nevertheless, the main lesson applicable to most LRS settings is that non-specialized health care providers can directly contribute to quality improvement in clinical care through specific antimicrobial stewardship activities.

In conclusion, we demonstrate that an enabling intervention that is tailored to existing systems is feasible with modest external support and training. Audit-feedback activities, with attention paid to bacteriology results, lead to significant decreases in antibiotic consumption and cost savings, and resulted in better outcomes than standard medical care in our setting. These findings should catalyze the commitment of the organizational leadership to sustain enabling antimicrobial stewardship activities and from health authorities to scale up similar programs in LRS.

## Data Availability Statement

The raw data supporting the conclusions of this article will be made available by the authors, without undue reservation, to any qualified researcher.

## Ethics Statement

The studies involving human participants were reviewed and approved by institutional review board, College of Health Sciences, Addis Ababa University. Written informed consent from the participants' legal guardian/next of kin was not required to participate in this study in accordance with the national legislation and the institutional requirements.

## Author Contributions

GG, MS, DH, and TG: conceptualization. MS and CY: funding acquisition. GG and AM: data collection. MS and WAb: project administration. GG and MS: data analysis and writing of first draft. DH, WAb, AM, WAm, TA, TB, ML, and CY: review and editing.

### Conflict of Interest

The authors declare that the research was conducted in the absence of any commercial or financial relationships that could be construed as a potential conflict of interest.

## Abbreviations

AMR: Antimicrobial resistance
AMS: Antimicrobial stewardship
DOT: Days of therapy
ID: Infectious diseases
LMICs: Low- and middle-income countries
LOS: Length of hospital stay
TASH: Tikur Anbessa Specialized Hospital
3GC: Third generation cephalosporins
4GC: Fourth generation cephalosporins.
