# Supplementary material for: Half of Prescribed Antibiotics Are Not Needed: A Pharmacist-Led Antimicrobial Stewardship Intervention and Clinical Outcomes in a Referral Hospital in Ethiopia
Source: Front Public Health. 2020 Apr 9;8:109. doi: 10.3389/fpubh.2020.00109 (PMC7160317; doi:10.3389/fpubh.2020.00109)
Supplement: Supplementary file 1 [file Data_Sheet_1.docx]

**ANTIMICROBIAL STEWARDSHIP TEAM RECOMMENDATIONS – Chart copy**

Patient name _______________________________________ MRN_____________________ Bed No.:__________

This chart was reviewed on: ____/______/________ (dd/mm/yyyy)

| **Antibiotic 1** (Name, dose and route): | |
| --- | --- |
| **Provenance of antibiotics:** ☐ Hospital ☐ Private | |
| **Recommendation** | **Reason** |
| ☐ Discontinue (If new suspicion of infection, septic workup and reassess based on results) | ☐ Indication/source not evident by history  ☐ Septic workup insufficient for suspected focus  ☐ investigations do not support diagnosis/source of infection  ☐ Treatment duration sufficient |
| ☐ Continue for _____ days and reassess | ☐ Suggest additional investigations: _____________________________ |
| ☐ Change  ☐ Dose/Frequency________________  ☐ Route ____________________  ☐ To: ______________________  (New antibiotic name and dose) | ☐ Dosing inappropriate: ____________________  ☐ Spectrum inappropriate: __________________  ________________________________________  ☐ Route inappropriate: _____________________  ________________________________________ |
| Recommendation of Antibiotic 1 | ☐ Accepted ☐ Not accepted |
| **Antibiotic 2:** (Name, dose and route): | |
| **Provenance of antibiotics:** ☐ Hospital ☐ Private | |
| **Recommendation** | **Reason** |
| ☐ Discontinue (If suspicion of infection, septic workup and reassess based on results) | ☐ Indication/source not stated  ☐ Septic workup insufficient  ☐ investigations do not support diagnosis/source of infection  ☐ Treatment duration sufficient |
| ☐ Continue for _____ days and reassess | ☐ Suggest additional investigations: ________________________________________ |
| ☐ Change  ☐ Dose/Frequency________________  ☐ Route ____________________  ☐ To: ______________________  (New antibiotic name, dose and route) | ☐ Dosing inappropriate: __________________  ☐ Spectrum inappropriate: ________________  ______________________________________  ☐ Route inappropriate: ___________________  _______________________________________ |
| Recommendation of Antibiotic 2 | ☐ Accepted ☐ Not accepted |
| **☐ Recommend to consult ID; reason for consult: ______________________________________________** | |
| Recommendations follow-up at 24-48h | ☐ Fully Accepted ☐ Partially Accepted ☐ Not Accepted  If Not/Partially Accepted; reason: ______________________ |

____________________________________________ _________________________________________________

Pharmacist ID physician
