# Supplementary material for: Half of Prescribed Antibiotics Are Not Needed: A Pharmacist-Led Antimicrobial Stewardship Intervention and Clinical Outcomes in a Referral Hospital in Ethiopia
Source: Front Public Health. 2020 Apr 9;8:109. doi: 10.3389/fpubh.2020.00109 (PMC7160317; doi:10.3389/fpubh.2020.00109)
Supplement: Supplementary file 2 [file Data_Sheet_2.docx]

| Patient Initials: ______________ | | | | | | | | | | | | | | | | | | | | | | Patient MRN: ___ ___ ___ ___ ___ ___ | | | | |
| --- | --- | --- | --- | --- | --- | --- | --- | --- | --- | --- | --- | --- | --- | --- | --- | --- | --- | --- | --- | --- | --- | --- | --- | --- | --- | --- |
| Was patient previously enrolled in audit? | | | | | | | | | | | | | | | | | □ Yes 🡺  □ No | | | | | If yes, previous subject ID: _____________ | | | | |
| **Demographic and clinical Information** | | | | | | | | | | | | | | | | | | | | | | | | | | |
|  | Age: ______ years ______months | | | | | | | | | | | | | |  | | | Gender: □ Male □Female | | | | | | | | |
|  | Ward? | | | □ Pediatrics  □ Medicine  □ Surgery | | | | □ ICU  □ Gyn/Obs | | | □ ED  □ Other | | | | *104.* | | | Specify the unit (e.g. C-7, B-7, C-8, B-8 etc.): ________________ | | | | | | | | |
|  | Presumed infection at ADMISSION? | | | | | | | | | | | | | | □ Yes □ No (IF NO Infection suspected go to 107) | | | | | | | | | | | |
| *106* | Indication for **current** antibiotics? | | | | | *a)*  *b)*  *c)* | □ Empiric 🡺(go to 108)  □ Suspected/Confirmed (go to 106c)  What is the suspected source? (refer to indications): __________(select best option, up to 2 indications) | | | | | | | | | | | | | | | | | | | |
| *107.* | IF **NO Infection** suspected at ADMISSION, what is the reason for admission? 🡺 | | | | | | | | | | | | | □ Surgical □ Medical Causes □ Delivery □ Cancer | | | | | | | | | | | | |
| *108* | Is **hospital associated infection** suspected? | | | | | | | | | | | | | □Yes □ No | | | | | | | | | | | | |
|  | Admission date: *dd*_______*mm______ yyyy*__________ | | | | | | | | | | | | | | |  | | | | | | | | | | |
|  | Discharge Date: *dd*_______*mm______ yyyy*________ | | | | | | | | | | | | | | |  | | | | *If death during hospitalization,* date of death: *dd*_____ *mm______ yyyy*_____ | | | | | | |
|  | Preexisting Comorbidities? | | | | | | | | | □ Yes 🡺 □ No | | | □ Liver disease  □ Renal disease □ HIV | | | | | | | | | | □ Malignancy  □ Other(specify): ______________ | | | |
|  | | |  | | | | | | | | | | | | | | | | | | | | | | | |
| 1. ANTIBIOTIC **USAGE AT TIME OF AUDIT** | | | | | | | | | | | | | | | | | | | Total Number ANTIBIOTICS: #_______________ | | | | | | | |
|  | | Antibiotic  (Name, route) | | | Prescribed dose  (mg) | | | | Frequency  of administration | | | Start date  *(dd-mm-yyyy*) | | | | | | | | | End date  *(dd-mm-yyyy*) | | | Duration of TX  # Days | Provenance  H=Hospital  P=Private | Total Cost  of  ABX (ETB) |
| 1. ABX-1 | |  | | |  | | | |  | | | ___ -___ -___ | | | | | | | | | ___ -___ -___ | | |  |  |  |
| 1. ABX-2 | |  | | |  | | | |  | | | ___ -___ -___ | | | | | | | | | ___ -___ -___ | | |  |  |  |
| 1. ABX-3 | |  | | |  | | | |  | | | ___ -___ -___ | | | | | | | | | ___ -___ -___ | | |  |  |  |
| 1. ABX-4 | |  | | |  | | | |  | | | ___ -___ -___ | | | | | | | | | ___ -___ -___ | | |  |  |  |
| 1. ABX-5 | |  | | |  | | | |  | | | ___ -___ -___ | | | | | | | | | ___ -___ -___ | | |  |  |  |

PI Signature: _________________________________ Date: _________________________

| **PREVIOUS ANTIBIOTICS:** | | | | | | | | |
| --- | --- | --- | --- | --- | --- | --- | --- | --- |
| Were Antibiotics taken at TASH within the previous 4 weeks (prev_AB): | | | | □ Yes 🡺  □ No  □ Unknown | If yes, how many: ______________ | | | |
|  | Antibiotic  (Name, route) | Dose (mg) | Frequency  of adminis-tration | Start date  *(dd-mm-yyyy*) | End date  *(dd-mm-yyyy*) | Duration of TX # Days (calculated) | Provenance  H =Hospital  P=Private | Total Cost  of  ABX (ETB) |
| 1 |  |  |  | ___ -___ -___ | ___ -___ -___ |  |  |  |
| 2 |  |  |  | ___ -___ -___ | ___ -___ -___ |  |  |  |
| 3 |  |  |  | ___ -___ -___ | ___ -___ -___ |  |  |  |
| 4 |  |  |  | ___ -___ -___ | ___ -___ -___ |  |  |  |

| **INTERVENTIONS SUGGESTED FOR THE CURRENT ANTIBIOTICS** | | | | | | | |
| --- | --- | --- | --- | --- | --- | --- | --- |
| **Antibiotic** | **AMS Team**  **Suggestion(s)** | | **If suggested to change or discontinue, what was the reason* (Write No. from below - all that apply)** | | | | **Acceptance:** |
| ABX-1 | □ Continue  □ Change (dose/freq/route)  □ Discontinue | |  | | | | □ Yes  □ No |
| ABX-2 | □ Continue  □ Change (dose/freq/route)  □ Discontinue | |  | | | | □ Yes  □ No |
| ABX-3 | □ Continue  □ Change (dose/freq/route)  □ Discontinue | |  | | | | □ Yes  □ No |
| ABX-4 | □ Continue  □ Change (dose/freq/route)  □ Discontinue | |  | | | | □ Yes  □ No |
| ABX-5 | □ Continue  □ Change (dose/freq/route)  □ Discontinue | |  | | | | □ Yes  □ No |
| **Additional recommendation:** | | □ **Add** Additional ABX | | | If YES🡺What ABX : ( as per list): ____________ | | |
|  |  |  |  |  | What is reason: (see list 6 or,9): _______________ | | |
|  |  | □ Consult ID | | | | | |
| **REASONS** | | | | | | | |
| Reasons for **Discontinuation ABX** | | | | Reasons for **Change ABX** | | Reasons for **Addition ABX** | |
| 1. Indication/source not evident by history  2. Septic workup insufficient  3. Investigations do not support Dx/source of infection  4. Treatment duration sufficient  6. Spectrum inappropriate | | | | 5. Dosing inappropriate  7. Route inappropriate  8. Safety considerations  10. Cost /Availability | | 6. Spectrum inappropriate or insufficient  9. Additional source suspected | |

| **Antibiotics** | | | |
| --- | --- | --- | --- |
| 1. Amikacin (iv/im)  2. Amoxicillin (po)  3. Amoxicillin+ClavulanicAcid (po) (Clavulin, Augmentin)  4. Ampicillin (iv/im)  5. Ampicillin (po)  6. Azithromycin (Zithromax) (iv)  7. Azithromycin (Zithromax) (po)  8. Cefepime (iv)  9. Cefazolin (Ancef) (iv/im)  10. Cefixime (Suprax) (po)  11. Cefotaxime (Claforin) (iv, im)  12. Cefoxitin (iv, im)  13. Ceftazidime (iv/im)  14. Ceftriaxone (iv/im)  15. Cefuroxime (iv/im)  16. CefuroximeAxetil (Ceftin)  17. Cephadroxil (po)  18. Cephalexin (cefalexin) (po)  19. Chloramphenicol (iv)  20. Chloramphenicol (po)  21. Ciprofloxacin (po)  22. Ciprofloxacin (iv)  23. Clarithromycin (Biaxin) (po)  24. Clindamycin (iv/im) | 25. Clindamycin (po)  26. Cloxacillin (iv)  27. Cloxacillin (po)  28. Doxycycline (iv)  29. Doxycycline (po)  30. Erythromycin (po)  31. Gentamycin (iv, im)  32. Imipenem + cilastatin (iv)  33. Moxifloxacin (po)  34. Meropenem (iv)  35. Metronidazole (Flagyl) (iv)  36. Metronidazole (Flagyl) (po)  37. Moxifloxacin (Avelox) (iv)  38. Nalidixic acid (po)  39. Ofloxacin (po)  40. Nitrofurantoin (po)  41. Norfloxacin (po)  42. Penicillin benzathine Benzyl) (im)  43. Penicillin G (iv)  44. (Benzyl) penicillin procaine (IM) | 45. Penicillin V Potassium(phenoxymethylpenicillin) (oral)  46. Piperacillin (iv, im)  47. Piperacillin + Tazobactam (Tazocin) (iv)  48. Rifampicin (po)  49. Spectinomycin (iv)  50. Streptomycin (iv, im)  51. Sulfadiazine (iv)  52. Sulfadiazine (po)  53. Primaxin (Imipenem) (iv)  54. Tetracycline (po)  55. Ticarcillin-Clavulinic acid (Timentin) (iv)  56. Trimethoprim Sulfamethoxazole (Bactrim, Septra)(iv)  57. Trimethoprim Sulfamethoxazole (Bactrim, Septra)(po)  58. Vancomycin (iv)  59. Other (specify)__________________  60.Ampicillin+Sulbactam (iv)  61.Amoxacillin+Clavulinic acid (iv)  62. Linozelide (po)  63.Linezolide (iv) | |
| **Indications** | | | |
| 1. Peri-operative (prophylaxis)  2. Respiratory infection (URTI, LRTI, etc. CAP. HAP)  3. CNS infection (Meningitis, encephalitis, brain abscess, epidural abscess, etc.)  4. GI infection (gastroenteritis, perforated bowel, intraabdomnial abscess, etc.)  5. Genitourinary infection (UTI, pyelonephritis, PID, prostitis, cystitis, endometritis)  6. MSK infection (osteomyelitis, septic arthritis, prosthetic infection etc.)  7. Cardiovascular infection (endocarditis, myocarditis, pericarditis etc.)  8. Systemic viral infection (dengue, VHF, etc.)  9. Skin/soft tissue infection (cellulitis, abscess, wound infection etc.) 10. Fever of unknown origin  11. Febrile neutropenia  12. HIV related | | | 13. Malaria  14. Relapsing fever  15. Visceral Leishmaniasis  16. Typhoid fever  17. Typhus (louse-borne)  18. Other specify: __________  19. Unspecified  20. LONS Neonatal sepsis  21. Aspiration Pneumonia  22. SSI- Surgical site infection  23. Line infection_IV site  24. Prophylaxis |
